# Anthracycline-Induced Cardiotoxicity: Molecular Insights Obtained from Human-Induced Pluripotent Stem Cell–Derived Cardiomyocytes (hiPSC-CMs)

**DOI:** 10.1208/s12248-021-00576-y

**Published:** 2021-03-14

**Authors:** William P. Bozza, Kazuyo Takeda, Wei-Lun Alterovitz, Chao-Kai Chou, Rong-Fong Shen, Baolin Zhang

**Affiliations:** 1 Office of Biotechnology Products, Center for Drug Evaluation and Research, Food and Drug Administration, Silver Spring, Maryland 20993 USA; 2 Microscopy and Imaging Core Facility, Center for Biologics Evaluation and Research, Food and Drug Administration, Silver Spring, Maryland 20993 USA; 3 High-performance Integrated Virtual Environment, Center for Biologics Evaluation and Research, Food and Drug Administration, Silver Spring, Maryland 20993 USA; 4 Facility for Biotechnology Resources, Center for Biologics Evaluation and Research, Food and Drug Administration, Silver Spring, Maryland 20993 USA

**Keywords:** anthracycline, cardiotoxicity, cellular model, hiPSC-CMs

## Abstract

Anthracyclines are a class of chemotherapy drugs that are highly effective for the treatment of human cancers, but their clinical use is limited by associated dose-dependent cardiotoxicity. The precise mechanisms by which individual anthracycline induces cardiotoxicity are not fully understood. Human-induced pluripotent stem cell–derived cardiomyocytes (hiPSC-CMs) are emerging as a physiologically relevant model to assess drugs cardiotoxicity. Here, we describe an assay platform by coupling hiPSC-CMs and impedance measurement, which allows real-time monitoring of cardiomyocyte cellular index, beating amplitude, and beating rate. Using this approach, we have performed comparative studies on a panel of four anthracycline drugs (doxorubicin, epirubicin, idarubicin, and daunorubicin) which share a high degree of structural similarity but are associated with distinct cardiotoxicity profiles and maximum cumulative dose limits. Notably, results from our hiPSC-CMs impedance model (dose-dependent responses and EC_50_ values) agree well with the recommended clinical dose limits for these drugs. Using time-lapse imaging and RNAseq, we found that the differences in anthracycline cardiotoxicity are closely linked to extent of cardiomyocyte uptake and magnitude of activation/inhibition of several cellular pathways such as death receptor signaling, ROS production, and dysregulation of calcium signaling. The results provide molecular insights into anthracycline cardiac interactions and offer a novel assay system to more robustly assess potential cardiotoxicity during drug development.

**Supplementary Information:**

The online version contains supplementary material available at 10.1208/s12248-021-00576-y.

## INTRODUCTION

Anthracyclines are a class of chemotherapies that are widely prescribed for the treatment of human cancers (1, 2), which include doxorubicin, epirubicin, idarubicin, and daunorubicin. These drugs share a high degree of similarity in their chemical structures but are associated with distinct profiles of cardiotoxicity, ranging from acute events to long-term cardiac damages. Acute cardiotoxicity causes cardiac beating abnormalities such as tachycardia, bradycardia, non-specific ST-T wave changes, and atrioventricular and bundle-branch block which can be detected by electrocardiogram (ECG). Delayed cardiotoxicity results in reduced left ventricular ejection fraction (LVEF) and/or congestive heart failure. The cardiotoxicity of anthracyclines is dose dependent, which is highlighted by the recommended cumulative dose limits for each anthracycline drug (Table I) (3, 4).

**Table I:** Anthracycline Drug Product Information

Drug	Infusion dosage (mg/m^2^)	Maximum recomended cumulative dose (mg/m^2^)	Indication
Doxorubicin	60–75 every 3 weeks	400–450	Variety of solid and hematological malignancies
Epirubicin	100–120 every 3–4 weeks	900	Axillary node tumor involvement following resection of primary breast cancer
Daunorubicin	30–45 daily for 3 days	400–550	Acute nonlymphocytic leukemia Acute lymphocytic leukemia
Idarubicin	10–12 daily for 3–4 days	150	Acute myeloid leukemia

Doxorubicin may induce cardiotoxicity through inhibition of topoisomerase II, oxidative stress, dysregulation of intracellular calcium signaling, mitochondrial impairment, and apoptosis (5, 6). Our lab has recently shown that death receptor signaling driven by p53 is an additional critical component of doxorubicin-induced cardiotoxicity (7, 8). While doxorubicin-induced cardiotoxicity has been extensively studied, the molecular mechanisms behind epirubicin, idarubicin, and daunorubicin are less well understood.

Animal models are commonly used in preclinical evaluation of drug cardiotoxicity, which are low throughput and exhibit species differences in cardiac structure and function (9, 10). Data derived from animal models may not always translate into humans (11). Failure to accurately predict cardiotoxicity from animal models can result in increased risk to humans participating in clinical trials (false negatives) or can lead to unnecessary drug attritions (false positives). Recently, human-induced pluripotent stem cell–derived cardiomyocytes (hiPSC-CMs) have emerged as a powerful tool to assess drug-induced cardiotoxicity in a physiologically relevant and high-throughput format (12–14). The use of hiPSC-CMs was able to identify unexpected drug-induced cardiotoxicities in humans which were not observed in preclinical animal studies (11, 15).

Here, we describe an assay system that couples hiPSC-CMs and cellular impedance measurement. The assay can detect functional alterations such as beating rate, beating amplitude, and cellular index of hiPSC cardiomyocytes mimicking physically relevant conditions. We evaluated the assay suitability by testing a panel of four anthracyclines (doxorubicin, epirubicin, idarubicin, and daunorubicin). All agents elicited cytotoxic effects in hiPSC-CMs in a dose-dependent manner, yielding differential EC_50_ values that agreed well with their clinical dose limits. Genomic analysis identified several dysregulated pathways that are conserved across anthracyclines, but the extent to which each pathway was activated or inhibited was product specific. Notably, anthracyclines displayed significant differences in their ability to enter into hiPSC cardiomyocytes which were associated with their distinct cardiotoxicity profiles.

## MATERIALS AND METHODS

### hiPSC-CMs and Study Drugs

Human-induced pluripotent stem cell–derived cardiomyocytes (iCell cardiomyocytes^2^) were obtained from Fujifilm Cellular Dynamics, Inc., utilizing a validated iCell differentiation protocol that consistently produces hiPSC-CMs of high quality and purity. The hiPSC-CMs were routinely monitored for uniform plating confluency and consistent beating profiles using the CardioExcyte 96 platform (8). Cell culture plates were coated with 50 μg/ml fibronectin solution (Sigma-Aldrich) overnight at 37 °C. hiPSC-CMs were seeded at 1.56 × 10^5^ cells/cm^2^ in plating media. After 4 h of incubation, plating medium was replaced with maintenance media and media exchange was repeated every 48–72 h. After 5 days, when the hiPSC-CMs reached a stable beating monolayer under microscopy, anthracycline was added to the culture followed by impedance measurement or other assays. Doxorubicin hydrochloride (Catalog #D1515), epirubicin hydrochloride (E9406), and idarubicin hydrochloride (I1656) were purchased from Sigma-Aldrich. Daunorubicin hydrochloride (1467) was from Tocris Bioscience.

### Cellular Impedance Assays

hiPSC-CMs (50,000 per well) were seeded onto CardioExcyte96 sensor plates (Nanion) and were cultured as described above. Cardiomyocyte beating profiles and cellular index were recorded in real-time at 20-s intervals using the CardioExcyte 96 impedance system (Nanion). After anthracycline addition (0–5 μM), impedance signals were recorded every 90 min for 48 h. Cellular index and contractility data were normalized to pre-treatment values and were plotted against time for each anthracycline dose. EC_50_ values were calculated by nonlinear curve fitting of dose response curves from triplicate experiments. The GraphPad log[inhibitor] *vs.* response equation (four parameters) was used.

### Total RNA Isolation and Quantification

The hiPSC-CMs were cultured for 5 days and treated with individual anthracycline at 500 nM for 48 h. The resultant cells were collected by cell-scraper and centrifugation, followed by RNA extraction using the miRNeasy Micro kit (Qiagen). RNA content was estimated by UV spectrophotometry (Thermo Fisher Scientific), and quality was assessed using an Agilent BioAnalyzer RNA Nano Kit. Only samples with a RNA integrity number (RIN) ≥ 8 were proceeded for further analyses.

### RNAseq Data Generation, Processing, and Analysis

RNA sequencing (RNAseq) was performed as previously described (8). The Illumina TruSeq Stranded Messenger RNA Library Prep Kit was used to construct strand-specific libraries for sequencing. Samples were sequenced on the Illumina NovaSeq 6000 system. Paired-end sequencing (2 × 101 cycles) was performed and base call images generated by the NovaSeq 6000 were converted to fastq files for data analysis. Reads quality was evaluated by the FastQC. For each sample, more than 45 million reads were aligned against the reference genome GRCH30 using the high-performance integrated virtual environment (HIVE) moderated by FDA core facility (16). The Reads Per Kilobase Million (RPKM) matrix files were generated and genes were annotated using the HIVE. Regularized linear discriminant analysis (RLDA) algorithm was used to normalize the data and determine differential expression among genes in the anthracycline-treated *versus* control groups. Gene transcripts with greater than 0 RPKMs per sample, with greater than twofold change in expression compared to control groups, and with a *p* value significance of < 0.05, as determined by a two-tailed paired *t*-test, were further analyzed. Ingenuity pathway analysis (IPA) was used to analyze entire dysregulated mRNA gene sets. Using the HIVE and R Heatmap function, principal component analysis (PCA) and hierarchical clustering were performed using RPKM values of genes identified in select IPA cardiotoxicity pathways. To be included in the analysis, mRNA gene expression had to be significantly dysregulated across the entire panel of anthracyclines. Hierarchical clustering heat maps were generated by taking the logarithm base 10 of RPKM + 1 values and scaling by row.

### Immunoblotting

Whole cell lysates from hiPSC-CMs were subjected to immunoblotting analysis for protein expression. Briefly, cells were cultured onto 6 well plates for 5 days and treated with 150 or 500 nM anthracycline for 48 h. The resultant cells were collected by centrifugation and cell pellets were lysed in RIPA buffer. Protein content was determined using Pierce BCA Protein Assay Kit (Thermo Fisher Scientific). Equal amounts (30 μg per lane) of total proteins were resolved by NuPAGE 4–12% gradient Bis-Tris gels (Thermo Fisher Scientific) and were transferred to PVDF membranes. Membranes were immunoblotted with specified antibodies and were visualized using Immobilon Western Chemiluminescent HRP Substrate (Millipore) and an ImageQuant LAS 4000 imager (GE). Monoclonal antibodies against DR4 (D9S1R), DR5 (D4E9), TNFR1 (C25C1), and S15 phospho p53 (16G8) were purchased from Cell Signaling Technology. Anti-Fas antibody (C-20) was purchased from Santa Cruz Biotechnology. Anti-p53 antibody conjugated to HRP (cat # HAF1355) was purchased from R&D systems. Anti-GAPDH antibody (2D4A7) was purchased from Novus. Horseradish peroxidase–conjugated goat anti-rabbit IgG1 (sc-2054) and goat anti-mouse IgG1 (sc2969) were purchased from Santa Cruz Biotechnology.

### Confocal Microscopy

hiPSC-CMs were cultured on a Nunc Lab-Tek II chambered cover glass (Thermo Fisher Scientific) at 1.56 × 10^5^ cells/cm^2^ to form a beating monolayer. hiPSC-CMs were treated with 5 μM anthracycline for 24 h and were imaged using a Leica SP8 confocal microscope (Leica Microsystems) with PLAPO objective lens 40× (NA1.3). An excitation laser line of 488 nm (Emission 550-650 nm) was used to detect anthracycline fluorescence.

For colocalization imaging, hiPSC-CMs were first incubated with 100 nM MitoTacker Deep Red (Thermo Fisher Scientific) for 30 min, a mitochondria marker, followed by treatment with 5 μM anthracycline for 2 h. Live cell imaging was performed using an LSM 510 Meta Confocal Microscope attached to an Axiovert 200 Inverted Microscope (Carl Zeiss). An oil immersion 40× objective lens was used for optical imaging. Excitation laser lines of 488 and 633 nm were respectively used to detect anthracycline and mitochondria.

### Time-Lapse Imaging

hiPSC-CMs were seeded onto CellCarrier Ultra 96 well black plates with an optically clear cycl olefin bottom (Perkin Elmer Inc.) to form beating monolayers. Upon addition of anthracycline (5 μM), cells were immediately imaged using the Cell Voyager CV8000 High Content Screening system (Yokogawa Electric Corp.). The Cell Voyager stage incubator is equipped with temperature and CO_2_ control allowing images to be captured every 10 min for 200 min. A 60× water immersion objective lens (NA 1.2) was used for confocal imaging with excitation at 488 nm (Em 600 ± 37 nm) to detect anthracycline fluorescence.

## RESULTS

### Impedance Measurement of Anthracycline-Induced Toxicity in hiPSC-CMs

**Fig. 1:**
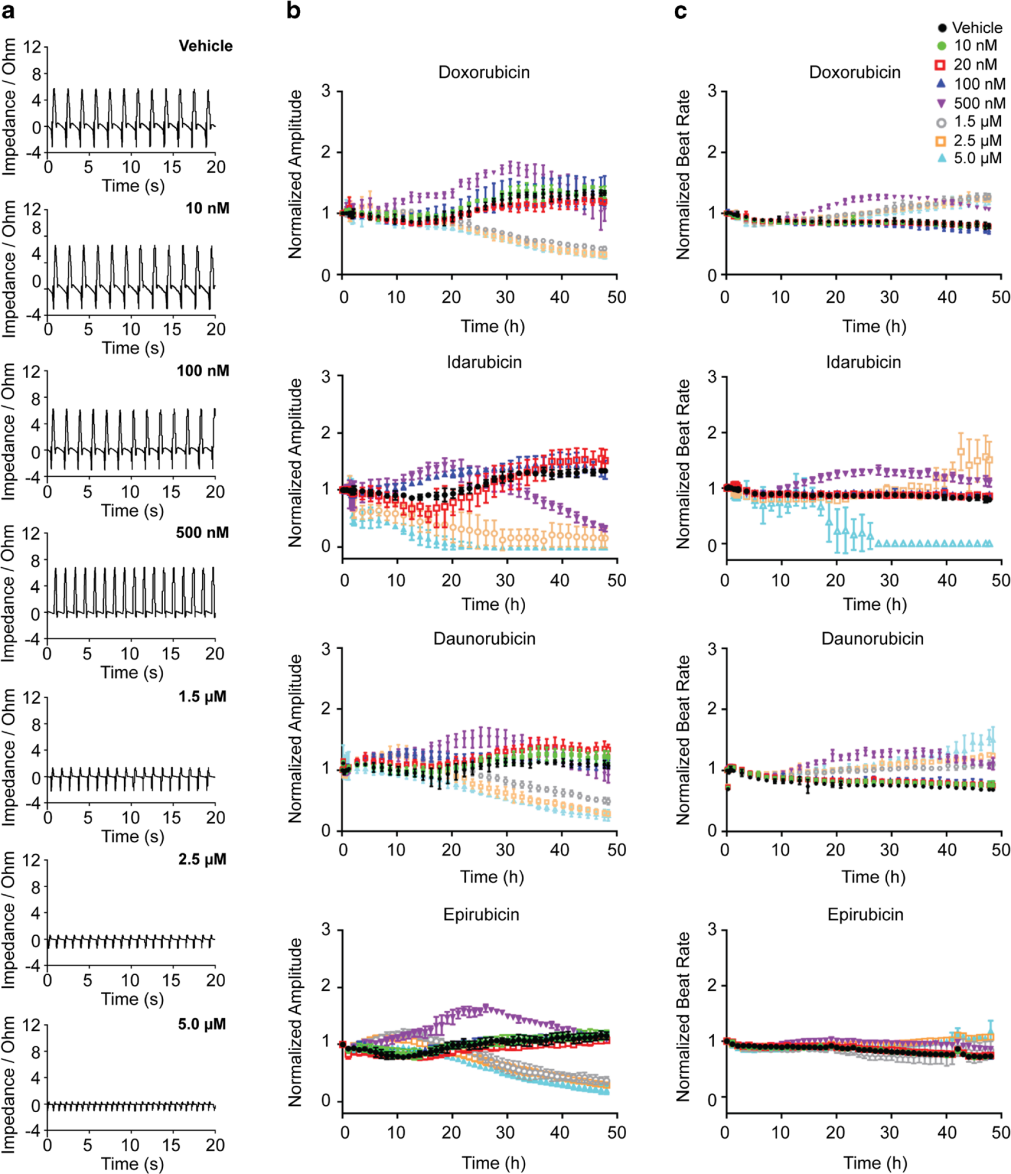
Anthracycline effect on cardiomyocyte beating. hiPSC-CMs were treated with anthracycline (0–5 μM) and impedance-based contractility measurements were recorded in real-time every 90 min for 48 h. **a** After 48 h, cardiomyocyte beating profiles displayed visible decreases in beating amplitudes and increases in beating rates. Normalized cardiomyocyte beating amplitude (**b**) and rate (**c**) were quantified and plotted *versus* time for each anthracycline treatment condition

**Fig. 2:**
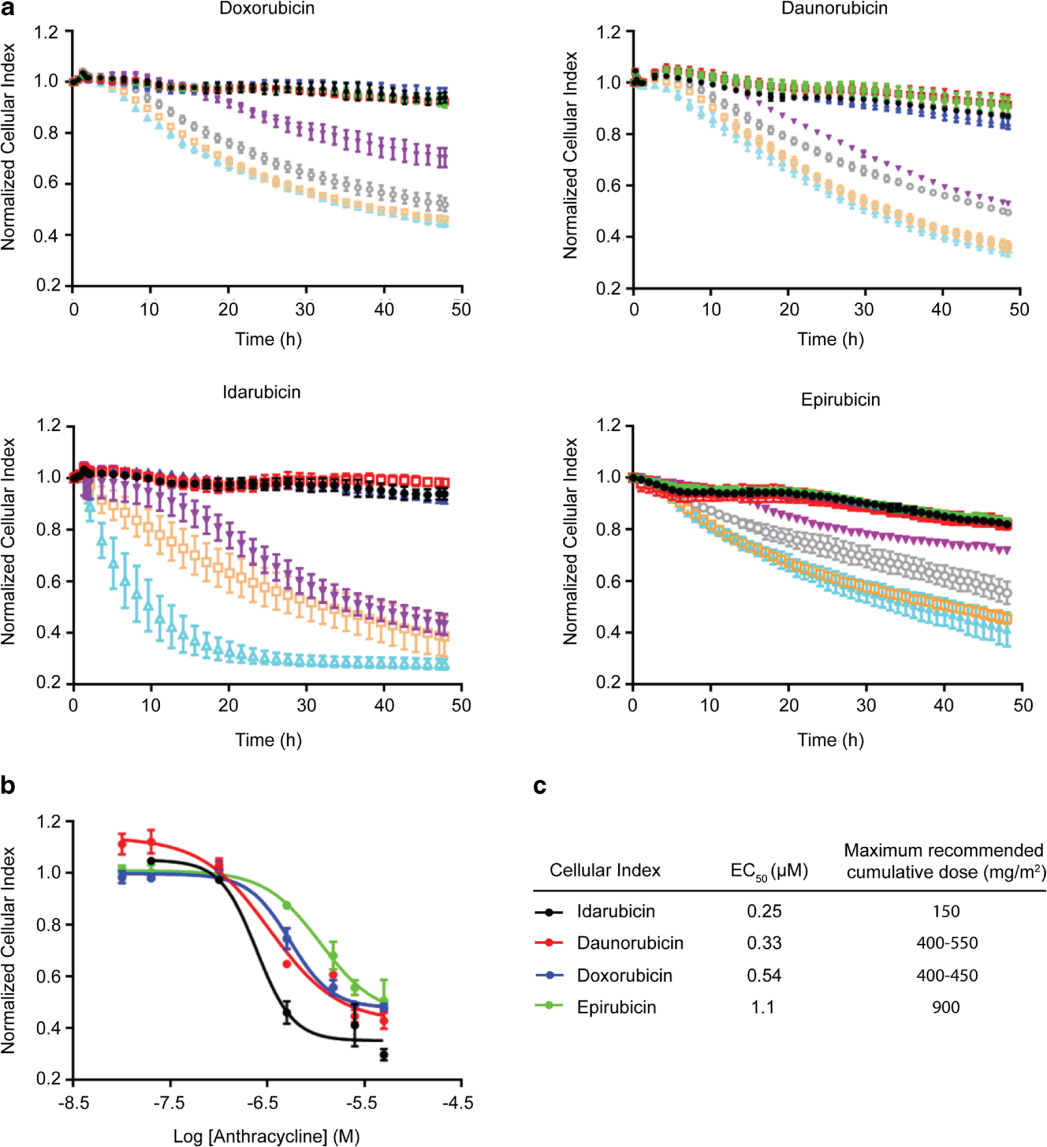
Anthracycline effect on cardiomyocyte cellular index. **a** Impedance-based cellular index of hiPSC-CMs was recorded in real time every 90 min for 48 h (vehicle = black, 10 nM = green, 20 nM = red, 100 nM = blue, 500 nM = purple, 1.5 μM = gray, 2.5 μM = orange, and 5.0 μM = cyan). **b** Normalized 48-h time point cellular index data were plotted *versus* log anthracycline concentration to generate dose response curves. **c** Dose response curves were fitted using nonlinear regression analysis to yield EC_50_ values. Notably, EC_50_ values agreed well with recommended maximum cumulative dose limits for those drugs

All anthracyclines caused dose-dependent alterations in beating profiles (Fig. 1a), which was accompanied by a decrease in beating amplitude (Fig. 1b) and a concomitant increase in beating rate (Fig. 1c). Idarubicin displayed the strongest effect and caused the hiPSC-CMs to completely stop beating at 5 μM. Cellular index decreased in a dose-dependent manner, reflecting composite effects on cell attachment, morphology, and viability (Fig. 2a). Dose response curves (Fig. 2b) were generated from 48-h time point data, yielding EC_50_ values (Fig. 2c). Notably, the results agreed with the maximum recommended clinical cumulative dose limits for each anthracycline (Fig. 2c and Table I). Clinically, idarubicin is associated with the lowest allowable cumulative amount (150 mg/m^2^) and was therefore anticipated to be the most toxic to hiPSC-CMs. Indeed, idarubicin was found to be the most cytotoxic agent among the tested drugs, displaying the lowest EC_50_ value (0.25 μM) in the hiPSC-CMs model. Epirubicin exhibited the greatest hiPSC-CMs EC_50_ value (1.1 μM), corresponding to the highest allowable cumulative clinical dose (900 mg/m^2^). EC_50_ values for daunorubicin and doxorubicin also followed the pattern of the recommended cumulative doses in cancer patients (Table I).

### Anthracycline-Induced Transcriptomic Alterations in hiPSC-CMs

**Fig. 3:**
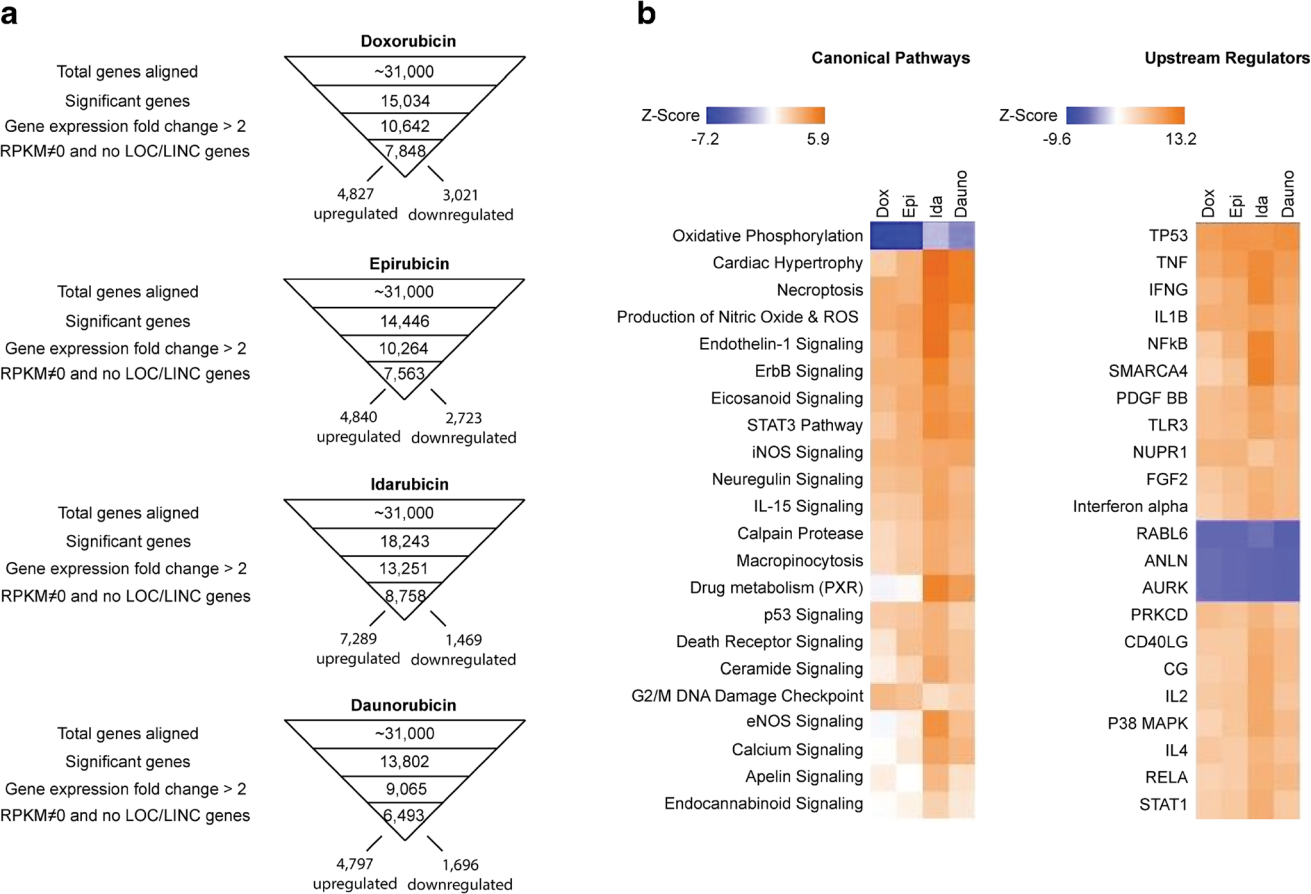
Transcriptomic analysis of anthracycline-induced cardiotoxicity. hiPSC-CMs were treated with 500 nM anthracycline for 48 h before RNA extraction and sequencing. **a** Regularized linear discriminant analysis (RLDA) algorithm was used to identify significant differential mRNA gene expressions for each anthracycline treatment compared to untreated control samples. Data sets were filtered using a *p* value significance < 0.05, fold-changes > 2, and RPKM ≠ 0. Only well-established gene transcripts were included (no LOC and LINC genes). **b** Ingenuity pathway analysis (IPA) algorithms were used to score canonical pathways and upstream regulator networks reported in literature based on inputted gene expression data. Activation *Z*-scores were shown using heat maps to compare each anthracycline

To gain insight into the molecular basis of anthracycline-induced cardiotoxicity, we assessed transcriptomic alterations in hiPSC-CMs by RNA sequencing (RNAseq). hiPSC-CMs monolayer cultures were treated with 500 nM anthracycline for 48 h. The assay conditions were chosen based on impedance results which showed a notable decrease in cellular index (Figs. 1 and 2). In addition, the same assay conditions (500 nM, 48 h) were used in our previous studies which demonstrated a consistent effect for doxorubicin on several signaling pathways (8). RNAseq results showed that many genes were significantly dysregulated (Fig. 3a) with the top 10 most downregulated consisting of cardiac structural genes (Figure S1) and the top 10 most upregulated genes relating to genotoxic stress, cannabinoid signaling, matrix metalloproteinase, and anti-angiogenesis (Figure S1).

Dysregulated mRNA gene sets were compared using ingenuity pathway analysis (IPA) (17). We highlighted several canonical pathways previously linked to doxorubicin-induced cardiotoxicity using heat maps, showing activation *Z*-score values (quantifies a match between predicted and observed expression patterns) for each anthracycline treatment condition (Fig. 3b, *left panel*). In addition, the top upstream regulators predicted to be activated based on mRNA gene expression changes are shown (Fig. 3b, *right panel*). This data demonstrates that the signaling pathways affected in hiPSC-CMs are well conserved across the panel of anthracyclines. However, idarubicin and daunorubicin displayed higher activation *Z*-scores compared to doxorubicin and epirubicin for many canonical pathways including ROS, calcium signaling, necroptosis, cardiac hypertrophy, and cardiac repair (apelin, ErbB, and endothelin-1 signaling). Doxorubicin and epirubicin displayed the highest activation *Z*-scores for G2/DNA damage checkpoints and inhibition of oxidative phosphorylation. This data suggests that each anthracycline drug can activate or inhibit specific cellular mechanisms to different extents.

**Fig. 4:**
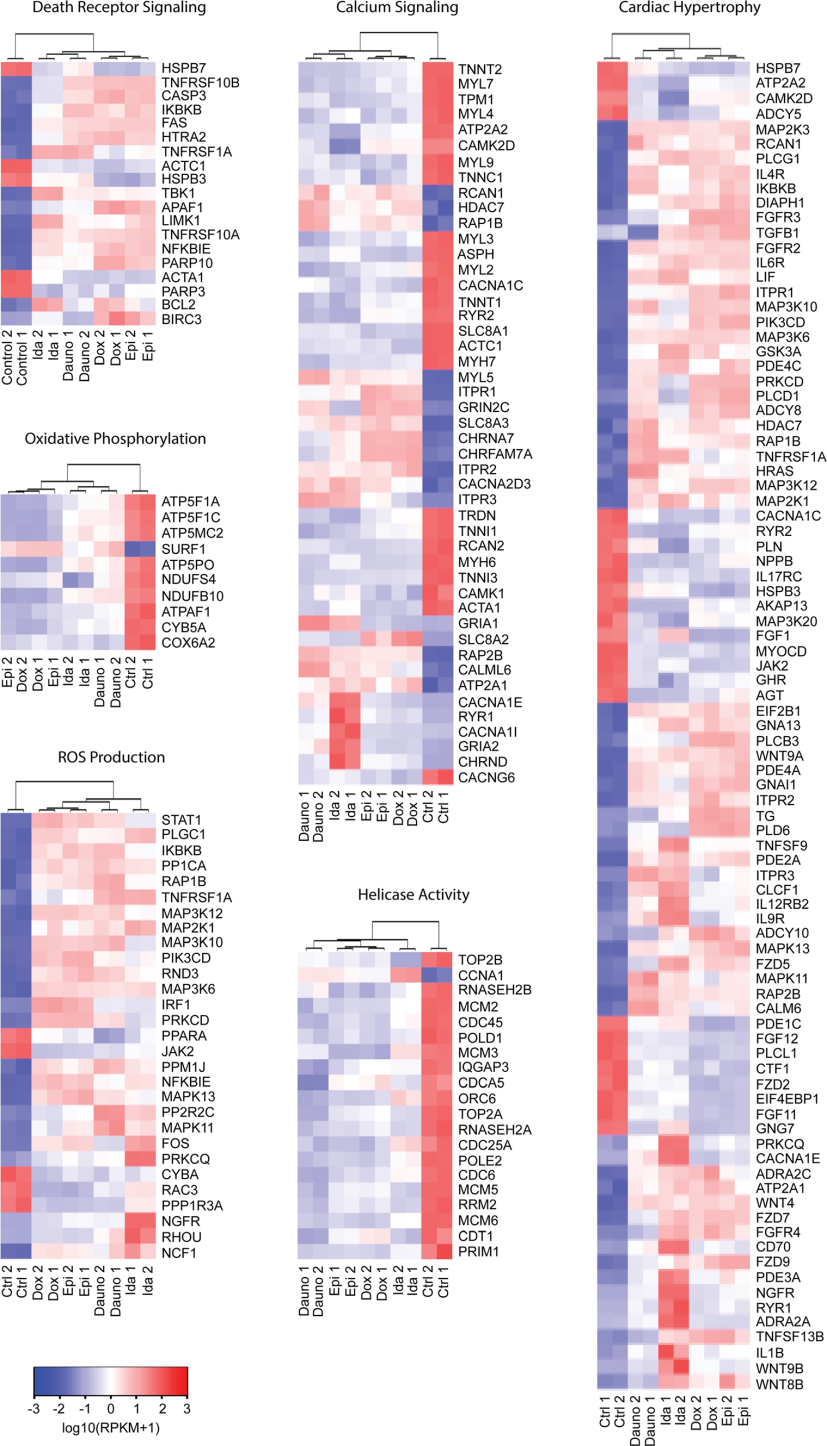
Hierarchical gene expression clustering of dysregulated cardiotoxicity molecular mechanisms. Heat mapping and hierarchical clustering were performed using RPKM values of dysregulated hiPSC-CMs genes identified in IPA cardiotoxicity pathways

**Fig. 5:**
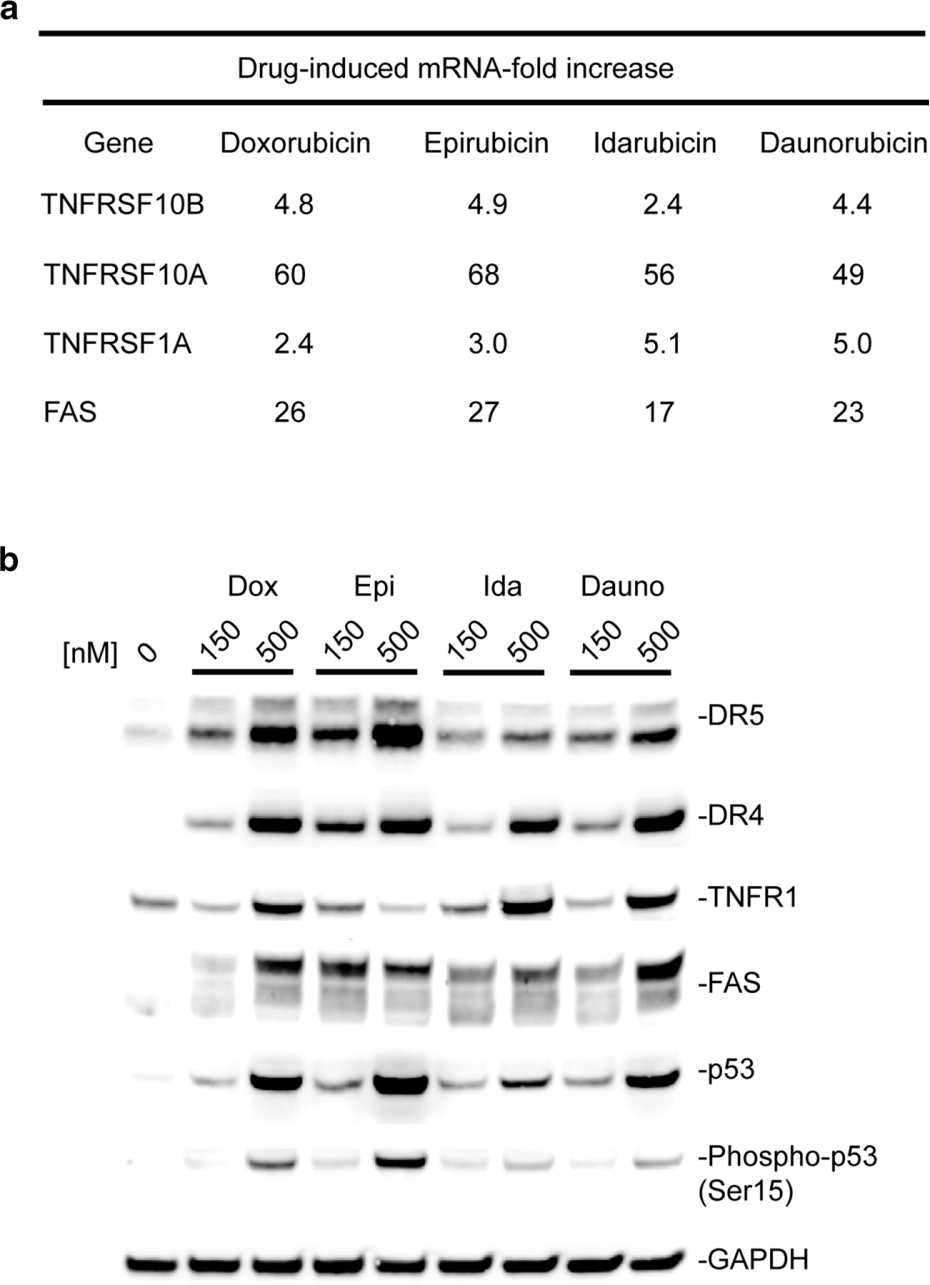
Upregulation of death receptor signaling as a critical component of anthracycline-induced cardiotoxicity. hiPSC-CMs were treated with 150 or 500 nM anthracycline for 48 h. **a** RNAseq results showing anthracyclines increased mRNA expression of death receptors in hiPSC-CMs. **b** Immunoblotting results confirmed anthracyclines induced cardiomyocyte overexpression of death receptors and p53 at the protein level

Principal component analysis (PCA) (Figure S2) and hierarchical clustering (Fig. 4) were performed using RPKM values of genes identified in relevant cardiotoxicity pathways. To be included in the analysis, mRNA gene expression had to be significantly dysregulated (fold-change > twofold and *p* value < 0.05) across the entire panel of anthracyclines. PCA and hierarchical clustering clearly demonstrate good replicate reproducibility and that anthracycline treatments clustered separately from vehicle controls. Interestingly, doxorubicin and epirubicin frequently clustered together and separated from idarubicin and daunorubicin which also clustered together. In hierarchical clustering data, this was observed for pathways of death receptor (DR) signaling, cardiac hypertrophy, calcium signaling, ROS, and oxidative phosphorylation (Fig. 4). We have previously shown p53-dependent upregulation of death receptor signaling as a critical component of doxorubicin-induced cardiotoxicity (7, 8). Similar observations were also made for epirubicin, idarubicin, and daunorubicin, which all upregulated expression of TNFRSF10B (DR5), TNFRSF10A (DR4), TNFRSF1A (TNFR1), and FAS (FAS) at mRNA (Fig. 5a) and protein levels (Fig. 5b). Immunoblotting data also serves to validate the observed changes of transcriptomic expression patterns in these genes.

### Dynamics of Anthracycline Cellular Uptake into hiPSC-CMs

**Fig. 6:**
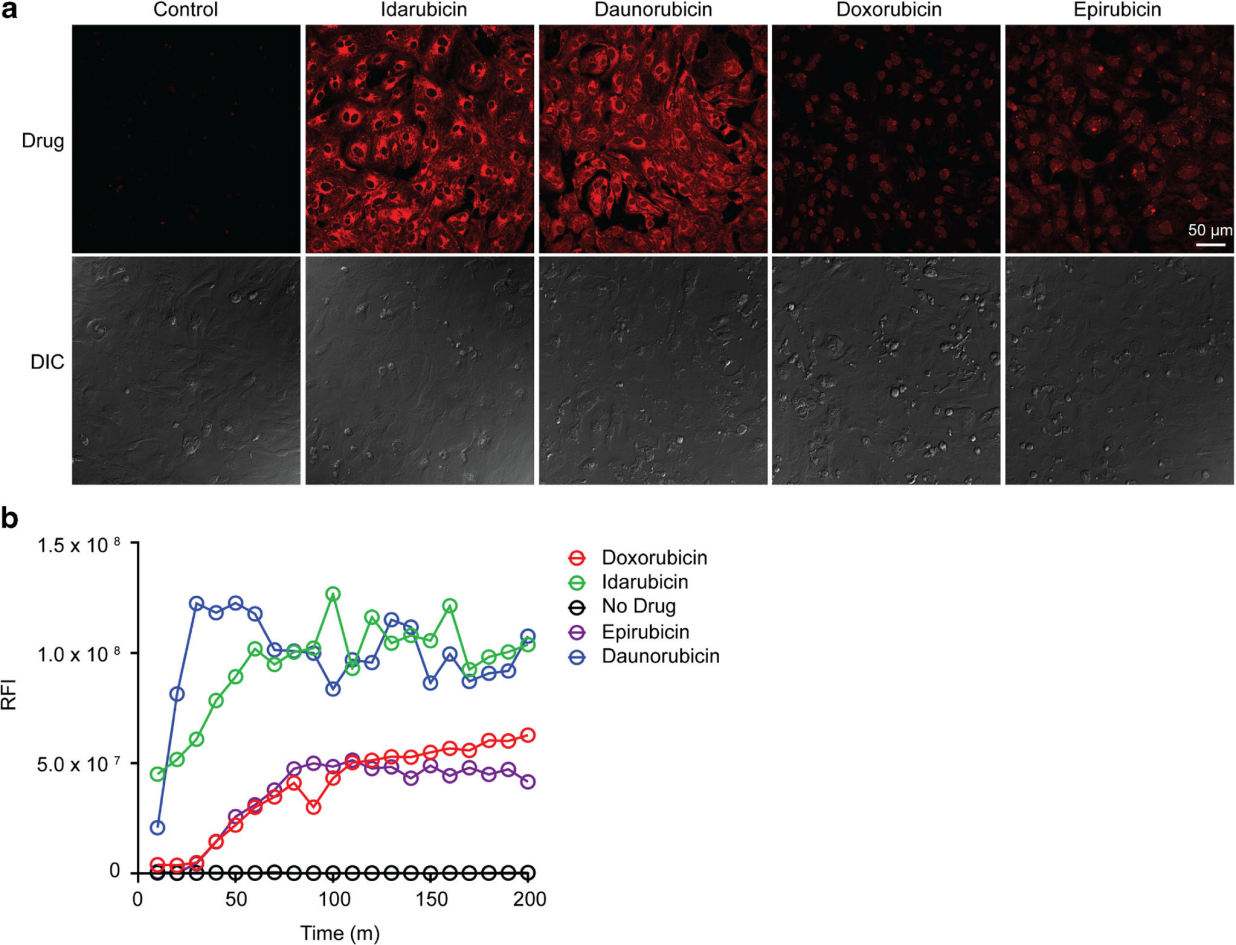
Dynamics of anthracycline cellular uptake. **a** Confocal microscopy images of hiPSC-CMs were taken after 24-h treatment with 5 μM anthracycline. Images were acquired using a 40× objective lens and laser excitation at 488 nm. Anthracycline fluorescence is shown in red. **b** Time-lapse imaging was performed every 10 min for 200 min. The RFI of representative cells was quantified and plotted *versus* time to compare anthracycline uptake rates. Shown are representatives of two replicates

Anthracyclines share a high degree of structural similarity; however, they differ in methoxy and hydroxyl groups rendering differences in lipophilicity (18) that may affect their cellular entry. We assessed the dynamics of anthracycline cellular uptake into hiPSC-CMs by exploiting the intrinsic fluorescence of each drug (Figure S3). In the cellular uptake assays, equal number of fully differentiated cardiomyocytes (which do not divide any further) were added to each well for analyses, resulting in the same cell density between samples. In addition, real-time live cell images were recorded over 200 min for each well. Together, this allows a fair comparison of cellular uptake across the panel of anthracyclines. Strikingly, confocal microscopy revealed the hiPSC-CMs cells absorbed significantly greater amounts of idarubicin and daunorubicin compared to doxorubicin and epirubicin (Fig. 6a). The real-time confocal images (Fig. 6) clearly show a profound preference in cardiomyocyte uptake for idarubicin and daunorubicin compared to doxorubicin and epirubicin. The data highlights that cardiomyocyte uptake is the first step towards explaining why idarubicin and daunorubicin are more cardiotoxic than epirubicin and doxorubicin. Notably, the observed differences of anthracyclines in cellular uptake into hiPSC-CMs were associated  with their distinct cytotoxicity patterns (Fig. 2) and clinical dose limits (Table I).

**Fig. 7:**
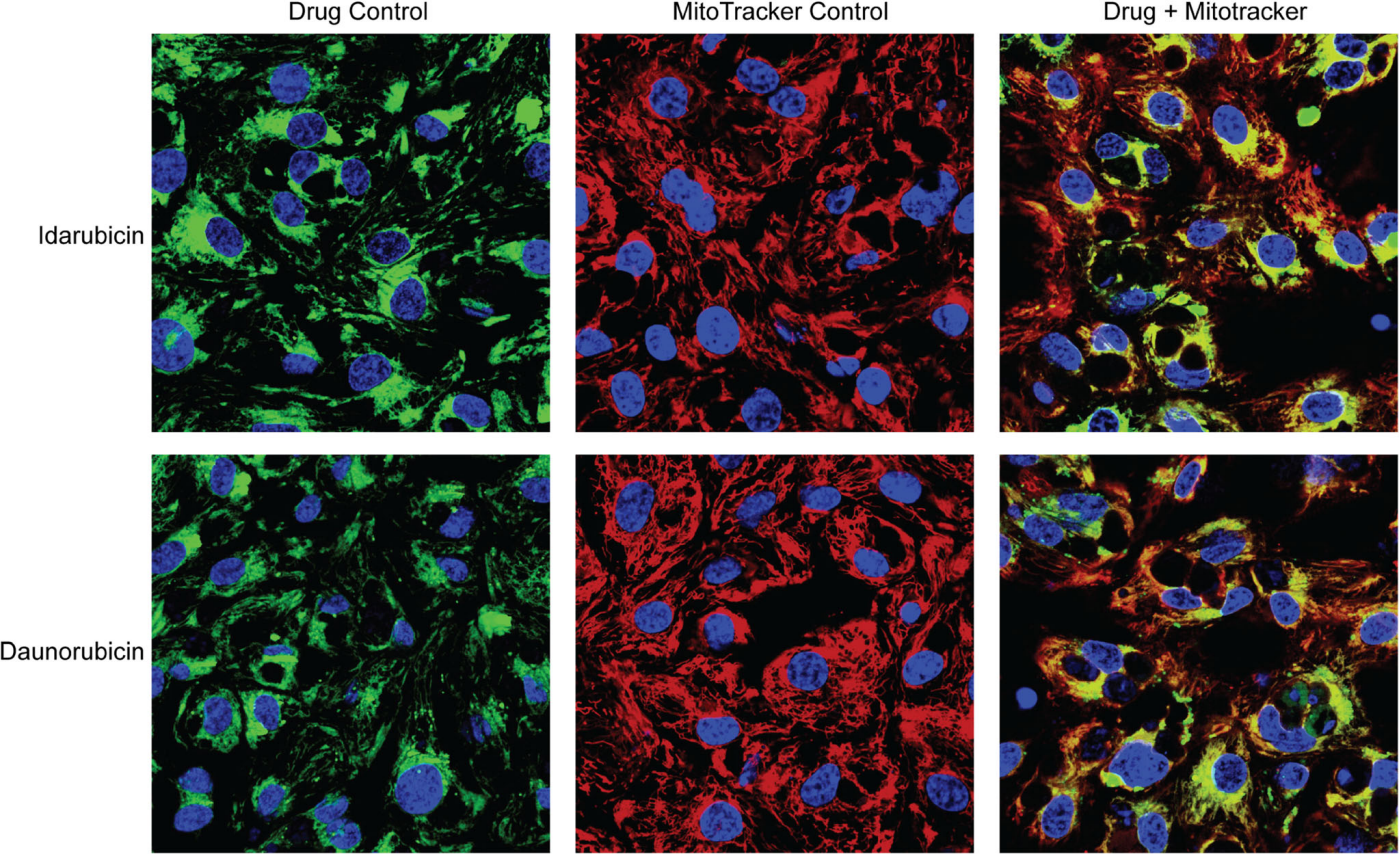
Mitochondrial localization of idarubicin and daunorubicin. hiPSC-CMs mitochondria were stained with Mitotracker Deep Red before treating with 5 μM idarubicin or daunorubicin for 2 h. Confocal microscopy images were acquired using a 40× objective lens and laser excitation at 405 nm for nucleus detection (shown in blue), 488 nm for anthracycline detection (shown in green), and 633 nm for mitochondria detection (shown in red). All images were acquired using the same red, blue, and green laser settings

Confocal microscopy analysis revealed that doxorubicin and epirubicin were primarily localized to the nucleus, while idarubicin and daunorubicin exhibited substantial non-nuclear localization (Fig. 6a). Anthracyclines are known to form irreversible complexes with cardiolipin in the inner mitochondrial membranes (19). We tested whether idarubicin and daunorubicin could be concentrating in the mitochondria due to their more lipophilic nature. Indeed, the result showed that idarubicin and daunorubicin were colocalized with mitochondria as visualized by staining with Deep Red MitoTracker (Fig. 7).

## DISCUSSION

We developed a novel assay system to assess drug cardiotoxicity utilizing physiologically relevant hiPSC-CMs coupled with cellular impendence measurement. After testing a panel of four anthracyclines, we show that the assay is robust in capturing functional attributes of hiPSC-CMs in response to drug treatment (Figs. 1 and 2). Such method offers several advantages over animal-based models and may be adopted as a preclinical model by pharmaceutical and academic institutions to better screen for potential cardiotoxicity during drug development.

Despite numerous efforts by the scientific community, the precise mechanisms by which anthracyclines induce cardiotoxicity are not fully understood. Previous studies on doxorubicin linked several cellular processes to its action in hiPSC-CMs (20). However, little is known about other anthracycline family members such as epirubicin, idarubicin, and daunorubicin. These anthracyclines share a high degree of structural similarity but are associated with distinct clinical dose limits (Table I). Using hiPSC-CMs, we were able to directly compare these drugs under the same assay conditions. The NGS samples were prepared under identical assay conditions from hiPSC-CMs after treatment with individual anthracycline drug at the same concentration (500 nM) and incubation time (48 h), which allows a fair comparison between the four drugs regarding their transcriptomic effects. The NGS data show a consistent pattern of dysregulation in several pathways across the panel of anthracyclines, but interestingly, to different extents for each drug. Our data identified several signaling pathways that are conserved in response to these drugs. To be highlighted, these include inhibition of helicase activity, increased intracellular calcium signaling, production of reactive oxygen species (ROS), death receptor signaling, reduced oxidative phosphorylation, cell death, and hypertrophy pathways (Fig. 3b). However, the extent to which each pathway is activated or inhibited is quite different between these drugs. Notably, idarubicin and daunorubicin induced much greater alterations in several gene clusters compared to doxorubicin and epirubicin (Figs. 3 and 4). The upregulation of death receptors in hiPSC-CMs may have an implication in identifying biomarkers to predict patients at higher risk to anthracycline-induced cardiotoxicity. These death receptors (TNFR1, Fas, DR4, and DR5) can induce apoptosis in target cells upon stimulation of their cognate ligand such as TNF, Fas ligand, and TRAIL. In light of the presence of TNF cytokines in circulation, whose serum levels can be very different among patients based on the status of disease and the history of treatment; we speculate that the elevated serum levels of TNF cytokines may be associated with a higher risk to anthracycline cardiotoxicity. Additional studies are warranted to evaluate the causal relationship between the baseline serum levels of specific TNF cytokines and the adverse cardiac events in cancer patients receiving anthracycline treatment.

We further showed a unique pattern of anthracyclines in cellular uptake into hiPSC-CMs. This was done by tracing the intrinsic fluorescence of anthracyclines (Figs. 6 and S3). Idarubicin and daunorubicin were found to be taken up by hiPSC-CMs at significantly higher rates and amounts compared to doxorubicin and epirubicin (Fig. 6). This is likely attributed to their higher lipophilicity from the subtle structural changes, daunorubicin lacks a hydroxyl group and idarubicin lacks a hydroxyl and a methoxy group compared to doxorubicin and its stereoisomer epirubicin. Together, the cellular uptake kinetics are in line with the NGS data which demonstrate key differences between the anthracyclines regarding their ability to enter cardiomyocytes and subsequently induce transcriptome changes.

## CONCLUSION

**Fig. 8:**
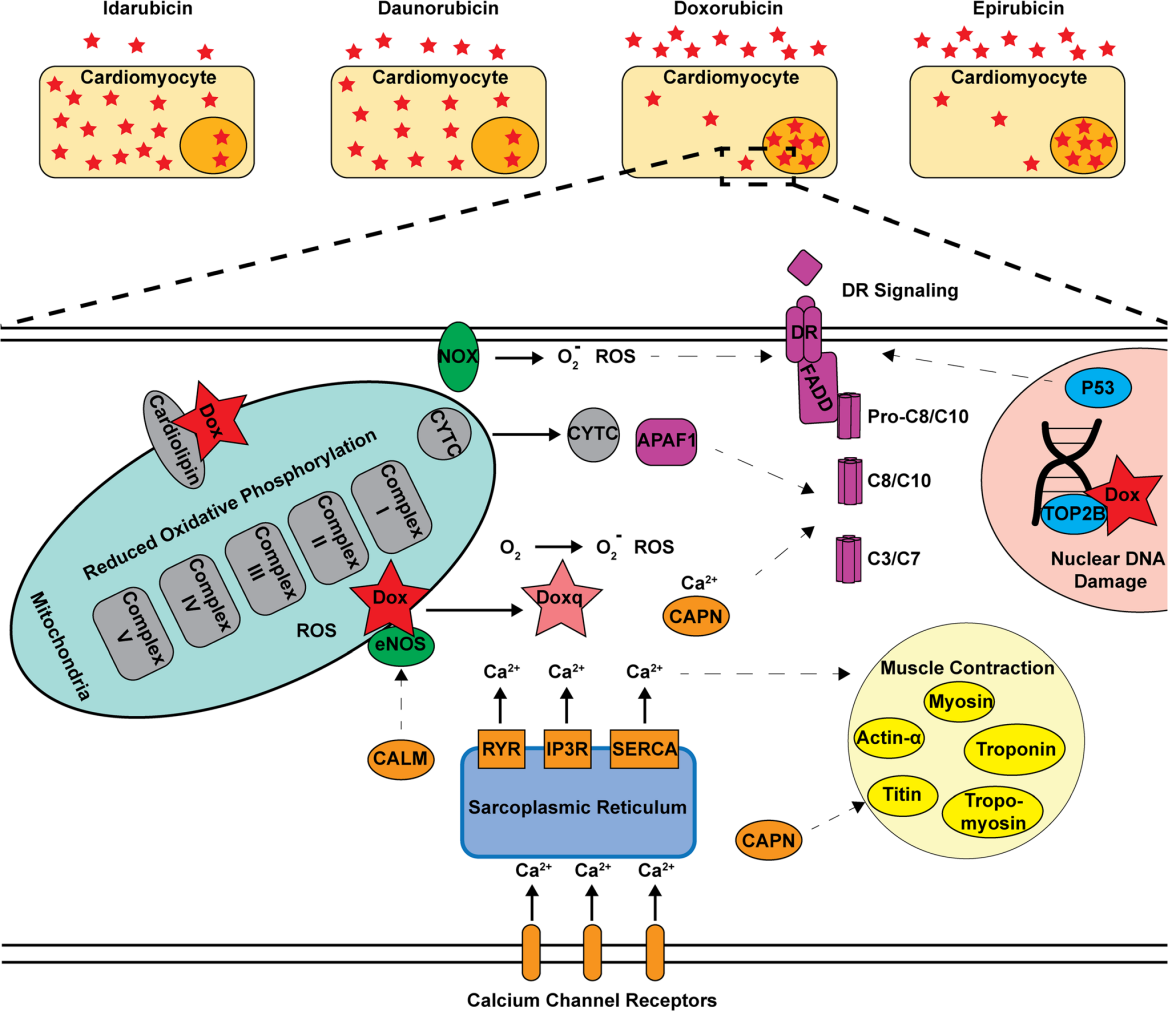
A mechanistic view of anthracycline-induced cardiotoxicity. Anthracyclines can be taken up by hiPSC-CMs at significantly different rates and amounts due to their differences in lipophilicity. Once inside the cell, they induce alterations in several inter-linked canonical pathways (dashed arrows) such as DR signaling (purple), DNA damage/helicase inhibition (blue), dysregulation of calcium signaling (orange), reduced oxidative phosphorylation (gray), ROS production (green), and cardiac structural damage (yellow). However, the extent to which each pathway is activated or inhibited is specific for each anthracycline drug and appears to be associated with the drug’s cellular uptake

Coupling hiPSC-CMs with impedance measurement offers a robust, high-throughput platform for assessing drug cardiotoxicity. The anthracycline family elicits dose-limiting cardiotoxicity through deregulation (inhibition or activation) of several conserved pathways in cardiomyocytes. The extent of pathway dysregulation was product specific and associated with their ability to enter hiPSC-CMs (Fig. 8).

## Supplementary Information

ESM 1 (DOCX 393 kb)
