# Supplementary material for: Anthracycline-Induced Cardiotoxicity: Molecular Insights Obtained from Human-Induced Pluripotent Stem Cell–Derived Cardiomyocytes (hiPSC-CMs)
Source: AAPS J. 2021 Mar 14;23(2):44. doi: 10.1208/s12248-021-00576-y (PMC7956936; doi:10.1208/s12248-021-00576-y)
Supplement: Supplementary file 1 — (DOCX 393 kb) [file 12248_2021_576_MOESM1_ESM.docx]

**Anthracycline-induced cardiotoxicity: Molecular insights obtained from human induced pluripotent stem cell-derived cardiomyocytes (hiPSC-CMs)**

**Supplementary Materials**


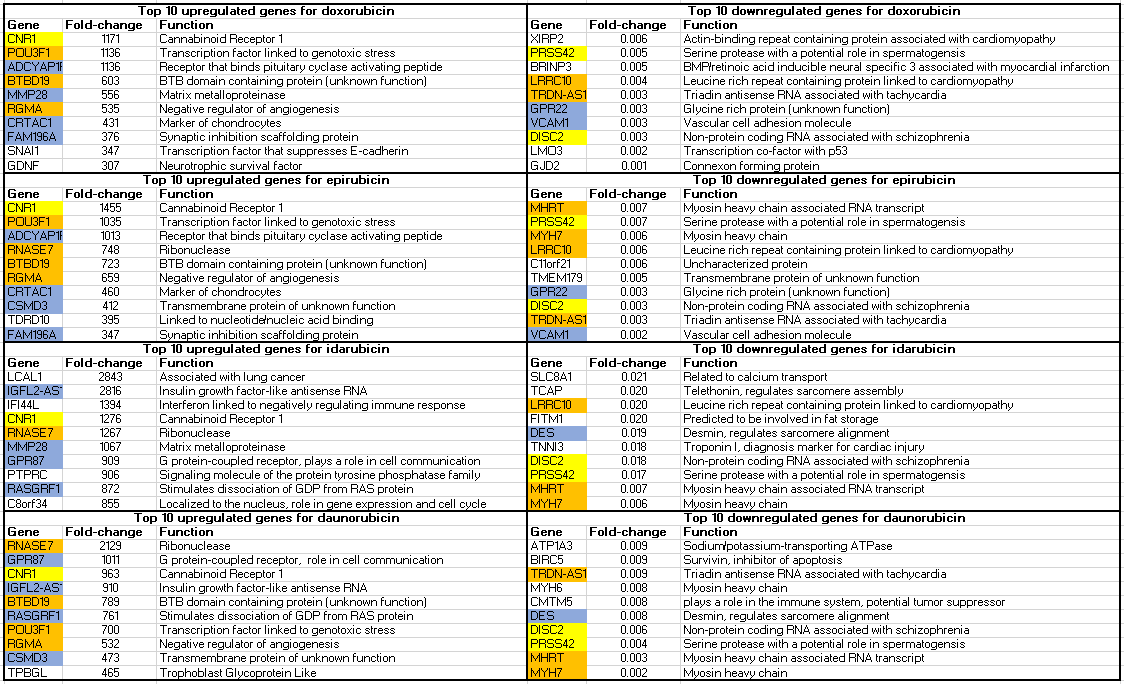


Figure S1. hiPSC-CMs were treated with anthracyclines (500 nM) for 48 hours before RNA extraction and sequencing. The top 10 most up- and downregulated genes are shown for comparison. Genes identified in the top 10 for all four anthracyclines are highlighted in yellow. Genes genes identified in the top 10 for three or two anthracyclines are highlighted orange and blue respectively.


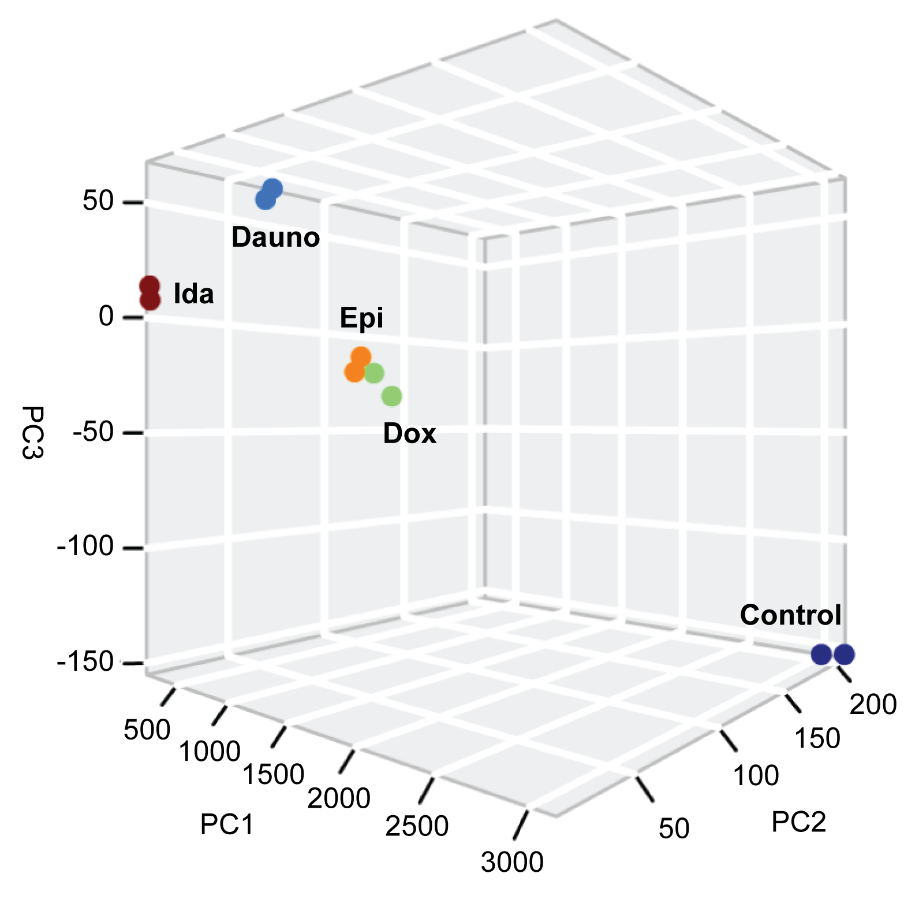


Figure S2. Principal component analysis (PCA) was performed using RPKM values of 177 genes identified in select IPA cardiotoxicity pathways.


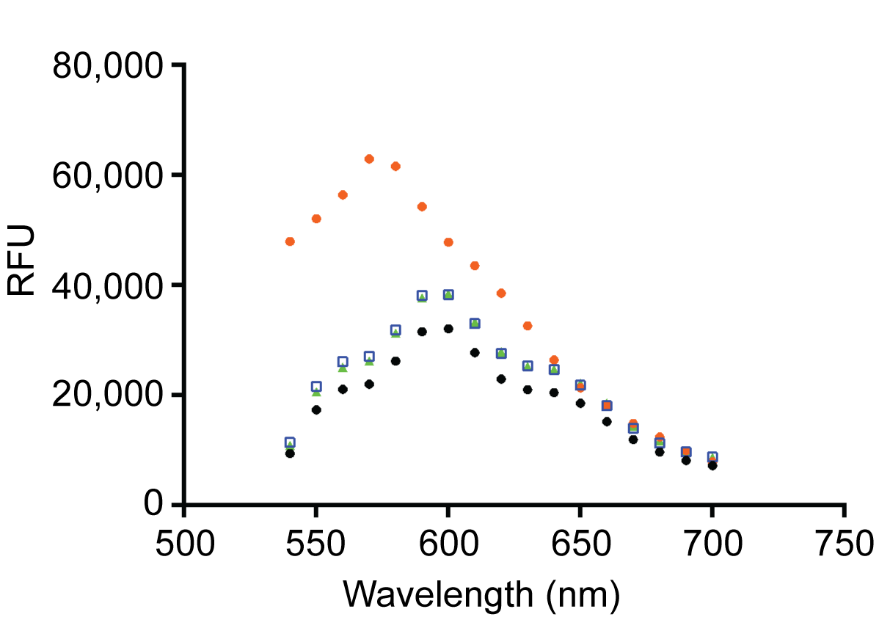


Figure S3. Fluorescence emission spectra of doxorubicin (black), epirubicin (green), idarubicin (orange), and daunorubicin (blue) when excited at 488 nm. Fluorescence was recorded using a BioTek microplate reader.
